# General Science

**Published:** 1869-05-15

**Authors:** 


### *General Science.*

The deplorable accident which occurred a short time ago in the Place de la Sorbonne, was, as our readers are probably aware, caused by an explosion of a quantity of powder destined to charge sub-marine torpedoes. Since that occurrence public attention, in France, has been intensely interested in the causes which brought it about, and the proper measures to be adopted to prevent the recurrence of similar accidents. The general press has given currency to the most erroneous reports of the origin of the explosion, and it is to set the matter right before the public, or at least, before that part of it best capable of judging of the matter, that we occupy your readers' attention with this subject. The non-scientific press universally stated that the accident was due to the spontaneous ignition of picrate of potash, a salt, according to their reports, which can scarcely be touched or heated without exploding instantly. This is a grave error which it is of moment to rectify, for we are persuaded that in no remote future, the picrates are destined to play an important part in industry. Picrate of potash is by no means the terrible salt represented. It can be struck or heated to 300 degrees without exploding. This fact is so well established that Mr. Designolle, inventor of a new powder, into the composition of which picrate of potash enters, has been anthorized to conduct his experiments in the Bouchet Powder Manufactory, one of the most important government mills. If the substance were as dangerous as the general press represents it, it is very unlikely that Mr. Designolle would have obtained this privilege. The quality which has so lately called attention to this salt of potash, is that it produces, when ignited, a large quantity of gas, and consequently possesses an enormous bursting power. To render its combustion complete, however, it must be mixed with nitrate or chlorate of potash, which burn the nascent carbon contained by the picric acid.

With nitrate of potash the combustion of the picrate is is slow, but nevertheless complete, a fact which permits its use as constituent of an ordinary gunpowder, which is much stronger than that composed of sulphur, charcoal and nitre. With chlorate of potash combustion is complete and instantaneous. So combined picrate of potash becomes an explosive powder absolutely unfit for fire arms, but excellent as a charge for bombs, and, above all, for the marine torpedoes. The instantaneous explosion of the mixture of picrate and chlorate of potash, a substance which, alone, can be hammered, and even pulverized in an iron mortar, with a pestle of seventy kilogrammes, as we have seen ourselves a hundred times. Chlorate of potash in combination with any organic substance, such as sugar or gum, is very easily exploded. It will be easily understood, then, that when mixed with an active substance such as picrate of potash, it becomes a very sensitive composition.

It is thus to the chlorate, and not to the picrate, of potash, that the mixture of these substances owes its peculiar susceptibility to explode, but it is the picrate which gives it the really fearful bursting power which it possesses.

